# The role of statins in dementia or Alzheimer’s disease incidence: a systematic review and meta-analysis of cohort studies

**DOI:** 10.3389/fphar.2025.1473796

**Published:** 2025-02-03

**Authors:** Ye Du, Zhangjie Yu, Chengyi Li, Yanxing Zhang, Buyun Xu

**Affiliations:** ^1^ Department of Neurology, Shaoxing People’s Hospital (Shaoxing Hospital, Zhejiang University School of Medicine), Shaoxing, China; ^2^ Department of Cardiology, Shaoxing People’s Hospital (Shaoxing Hospital, Zhejiang University School of Medicine), Shaoxing, China; ^3^ School of Medicine, Shaoxing University, Shaoxing, China

**Keywords:** Alzheimer’s disease, cohort study, dementia, statin, meta-analysis

## Abstract

**Background:**

The effect of statins on the risk of dementia and Alzheimer’s disease (AD) is unclear.

**Methods:**

We systematically searched EMBASE, Web of Science, PubMed, CENTRAL and ClinicalTrail.gov for cohort studies comparing incidence of new-onset dementia and AD between statin users and non-users. We applied the DerSimonian–Laird random effects method to pool hazard ratio (HR) with 95% confidence intervals (CI).

**Results:**

We included forty-two studies comprising 6,325,740 patients. Thirty-five cohort studies involving 6,306,043 participants were pooled and indicated that statin use was associated with a reduced risk of dementia (HR: 0.79, 95% CI: 0.71–0.88). Similarly, an analysis of 19 studies comprising 1,237,341 participants demonstrated a 29% decrease in the risk of AD among statin users (HR: 0.71, 95% CI: 0.60–0.85). In sensitivity analyses, diagnostic criteria for dementia/AD significantly affected the combined risk estimates. In subgroup analyses, compared to studies enrolling participants with a mean/median age over 70 years, those younger than 70 years exhibited greater efficacy of statins in preventing dementia (HR: 0.67, 95% CI: 0.56–0.81 vs HR: 0.86, 95% CI: 0.78–0.95; P = 0.02) and AD (HR: 0.47, 95% CI: 0.44–0.50 vs. HR: 0.81, 95% CI: 0.71–0.92; P < 0.01). Due to significant heterogeneity in the definitions of statin dosage and exposure duration, pooling the results was abandoned and most studies suggested that higher dosages and longer exposure duration of statins further reduce the risk of dementia and AD.

**Conclusion:**

Statin use is associated with a reduced incidence of dementia and AD, which might be modified by ages.

## 1 Introduction

Dementia is a chronic and progressive condition characterized by cognitive decline and impaired daily functioning, affecting approximately 50 million people globally (Mundal et al., 2022). As the global population ages, the prevalence of dementia is predicted to triple to 153 million in 2050 (Avan and Hachinski, 2023). Alzheimer’s disease (AD) is the most common form of dementia, followed by vascular dementia, Lewy body dementia, frontotemporal dementia and other forms (Mundal et al., 2022).

Statins, or HMG-CoA reductase inhibitors, are a group of medications commonly used to lower cholesterol levels and decrease the risk of cardiovascular diseases. Given the established link between cardiovascular health and cognitive function, there has been considerable interest in the potential neuroprotective effects of statins (Goldstein et al., 2023). In addition, several pleiotropic mechanisms have been proposed to explain the influence of statins on the risk of dementia, including anti-inflammatory and antioxidant effects, and improved cerebral blood flow (Olmastroni et al., 2022).

On the basis of these promising hypotheses, statins have been suggested to have a protective role against dementia. Several previous meta-analyses have reported that statin could reduce the risk of dementia (Olmastroni et al., 2022; Chu et al., 2018). However, subsequent cohort studies published after these meta-analyses have shown mixed results (Ren et al., 2024; Kim et al., 2023; Rajan et al., 2024; Zhou et al., 2021; Pham et al., 2022). Moreover, a recent Mendelian randomization study reported adverse effects of statins on cognitive function (Rosoff et al., 2022). This inconsistency may be attributed to variations in study design, population characteristics, types of statins used, dosages, and duration of follow-up. To address these uncertainties, an updated meta-analysis is warranted to synthesize the current evidence and offer a clearer understanding of the relationship between statins and dementia.

This meta-analysis aimed to systematically review and quantitatively analyze the available data from cohort studies to determine whether statin use is associated with a reduced risk of dementia, and if so, to what extent.

## 2 Methods

This systematic review and meta-analysis was conducted in accordance with the PRISMA (Preferred Reporting Items for Systematic Reviews and Meta-Analyses) guidelines. As the analyses were based solely on previously published studies, no ethical approval or informed consent was required. Supplementary Table S1 presents the PRISMA check list.

### 2.1 Search strategy and eligibility criteria

A comprehensive literature search was performed using EMBASE, Web of Science, PubMed, CENTRAL and ClinicalTrail.gov to identify eligible articles from their inception until 1 May 2024. The search strategy employed a combination of terms relevant to the research question, such as “statin”, “Alzheimer”, and “dementia”, along with suitable Boolean operators. The search was limited to full-text articles in English. A detailed search strategy is provided in Supplementary Table S2.

In accordance with PRISMA guidelines, all records obtained from the search were systematically screened based on their titles and abstracts. Articles that passed the abstract screening phase were then independently evaluated for full-text eligibility. Any disagreements during the screening process were resolved through discussion with a third author.

We included studies that met the following criteria based on the PICOS format: (1) Participants: individuals age over 18 years; (2) Intervention and comparison: statin users versus non-users; (3) Outcome: studies that reported an adjusted estimate (risk ratio [RR] or hazard ratio [HR]) and 95% confidence intervals (CIs) for the risk of all-cause dementia and/or AD. (4) Study design: observational cohort studies only. Additionally, the reference lists of the included studies were reviewed for other relevant articles that were not retrieved in the initial literature search. If multiple studies were based on the same cohort and investigated the same outcomes, only those with the largest sample sizes were selected for the meta-analysis.

### 2.2 Definition

Statin use was defined as ever used, continuously used, or initially used during the study and statin non-use was defined as never having used statins. The primary outcomes included all-cause dementia and AD, as defined by various clinical criteria. These criteria encompassed guidelines from the United States National Institute of Neurological and Communicative Disorders and Stroke and the Alzheimer’s Disease and Related Disorders Association, the International Classification of Diseases, 9th and 10th revisions (ICD-9/ICD-10), the American Psychiatric Association’s Diagnostic and Statistical Manual of Mental Disorders, as well as the Mini-Mental State Examination and its modified version.

### 2.3 Data extraction and quality assessment

Two authors independently extracted data from the eligible studies. The following data were extracted from each included study: the year of publication, sample size, patient characteristics, statin dosage and exposure duration, diagnostic criteria for dementia/AD and incidence of outcomes.

Two reviewers independently evaluated risk of bias in the studies, utilizing the New castle-Ottawa scale, which evaluates three domains: selection, comparability, and outcome. All results were cross-checked, and any disagreements were resolved through discussion. Studies with scores of 0–5, 6–7, and 8–9 were considered to have a high, medium, and low risk of bias, respectively.

### 2.4 Statistical analysis

Outcomes from the original studies were synthesized and compared using the DerSimonian–Laird random effects method. The effect measures are presented as HRs with 95% CIs. Heterogeneity was evaluated using Cochran’s Q statistic and the I2 metric. I^2^ values >50% indicated significant heterogeneity.

Sensitivity analyses were performed to evaluate the robustness of the pooled estimates and to examine the influence of individual studies on the pooled results and interstudy heterogeneity. Sensitivity analyses excluded studies with (1) a medium or high risk of bias (New castle-Ottawa scale < 8), (2) a sample size <10,000 patients, (3) publication before 2010, (4) inclusion criteria for participants other than the age and 5) non-ICD diagnostic criteria for dementia and AD. The p-values were determined by testing the homogeneity of the incidence of new dementia/AD between the included and excluded studies. In addition, to evaluate the effects of individual studies on the results, sensitivity analyses were performed by leave-one-out cross-validation.

Subgroup analyses were conducted to identify factors related to the incidence of outcomes. These subgroups were based on the mean/median age of the participants; sex; territory; and statin lipophilicity, dosage and exposure duration. The p-values for the subgroup analyses were determined by testing homogeneity.

To assess publication bias, we visually examined funnel plots depicting the standard error against the effect size and conducted Egger’s tests.

A two-sided p-value of <0.05 was considered significant. Statistical analyses were performed using the meta/metafor package in R statistical software (version 4.0.1, Vienna, Austria).

## 3 Results

**FIGURE 1:**
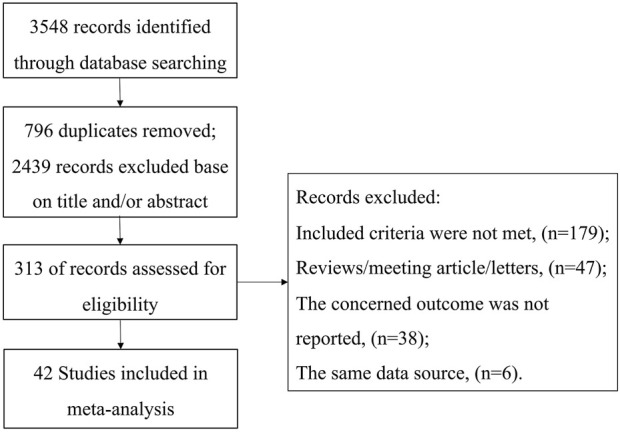
Flow diagram of the selection process of eligible articles.

The study selection process is depicted in Figure 1. Forty-two studies, involving 6,325,740 patients, were included in this meta-analysis. The main characteristics of these studies are summarized in Table 1. Of the included studies, 17 were conducted in Asia, 19 in North America, and 6 in Europe. Twenty-two studies focused on patients with specific comorbidities or characteristics. With the exception of 12 studies, the mean or median age of participants was over 70 years in all other studies. Seven studies exclusively reported AD as the outcome, 23 focused solely on dementia, and the remaining studies reported the incidence of both dementia and AD. The risk-of-bias assessment indicated that 17 studies had a low risk of bias, 24 had a medium risk, and one study had a high risk of bias. Details of the risk-of-bias assessment are summarized in Supplementary Figure S1.

**TABLE 1:** Characteristics of studies included.

Study	N	Including criteria	Age (year)	Male (%)	Territory	F.U. (year)	Outcomes	Diagnostic test
Li et al. (2004)	2,356	≥65 years	Mean (SD): 75.1 (6.1)	40.2	United States	6–8	D	DSM-IV, NINCDS-ADRDA
Reitz et al. (2005)	1,168	≥65 years	Mean (SD): 78.4 (6.2)	33.3	United States	4.8 ± 2.9	AD	NINCDS-ADRDA
Rea et al. (2005)	2,798	≥65 years	Mean: 75	40.1	United States	Mean: 3	D, AD	NINCDS-ADRDA
Zandi et al. (2005)	5,092	≥65 years	NR	45.0	United States	Mean: 3	D, AD	DSM-IV, NINCDS-ADRDA, 3MSE
Szwast et al. (2007)	1,141	≥65 years	Mean (SD): 73.3 (5.3)	30.7	United States	Mean: 3	D	DSM-III, ICD-10
Wolozin et al. (2007)	1,290,071	≥65 years Veterans	Mean (SD): 74.6 (5.6)	94.4	United States	NR	D	ICD-9
Arvanitakis et al. (2008)	929	>40 years	Mean (SD): 72.7 (6.1) in statin users; 75.2 (7.1) in nonusers	31.3	United States	<12	AD	MMSE
Cramer et al. (2008)	1,674	≥60 years	Mean (SD): 69.6 (6.2) in statin users; 70.4 (7.0) in nonusers	41.7	United States	Mean: 5	D	DSM-IV, NINCDS-ADRDA, 3MSE
Sparks et al. (2008)	2068	≥70 years with family history of AD	Mean (SD): 74.5 (3.6) in statin users; 74.9 (3.9) in nonusers	45.7	United States	NR	AD	DSM-IV
Haag et al. (2009)	6,992	≥55 years	Mean (SD): 69.4 (9.2)	40.0	Europe	Mean: 9.2	AD	DSM-III, NINCDS-ADRDA
Smeeth et al. (2009)	729,529	NR	NR	50.3	Europe	Median: 4.4	D, AD	Clinical chart record
Hippisley-Cox and Coupland (2010)	2,004,692	30–84 years	Mean (SD): 57.2 (11.7) in statin users; 44.4 (13.7) in nonusers	45.8	Europe	Median: 4.1	D	Clinical chart record
Li et al. (2010)	3,099	≥65 years	Mean (SD): 74.2 (5.5) in statin users; 75.8 (6.4) in nonusers	40.5	United States	Mean: 6.1	AD	DSM-IV, NINCDS-ADRDA
Beydoun et al. (2011)	1,604	>18 years	Mean (SD): 57.6 (9.2)	61.5	United States	Median: 25	D	DSM-III, NINCDS-ADRDA
Ancelin et al. (2012)	6,830	≥65 years	Mean (SD): 73.7 (10.5)	39.7	Europe	Mean: 7	D, AD	DSM-IV, NINCDS-ADRDA
Bettermann et al. (2012)	3,069	≥75 years	Mean (SD): 78.6 (3.3)	53.8	United States	Mean: 6	D, AD	3MSE, ADAS-Cog
Chen et al. (2014)	18,170	>50 years with diabetes mellitus	Mean (SD): 65.8 (7.4) in statin users; 67.0 (8.8) in nonusers	52.3	Asia	statin users: 3.9 ± 2.0; nonusers: 5.2 ± 3.1	D, AD	ICD-9
Chou et al. (2014)	33,398	≥60 years	NR	46.1	Asia	5.05 ± 5.36	D, AD	ICD-9
Ma et al. (2014)	634	≥65 years with mild cognitive impairment and diabetes mellitus	Mean (SD): 75.3 (5.9)	0.0	Asia	4.2 ± 1.2	AD	NINCDS-ADRDA
Chao et al. (2015)	256,265	≥60 years with atrial fibrillation	Mean (SD): 73.2 (7.4)	49.7	Asia	Maximum: 10	D	ICD-10
Chitnis et al. (2015)	8,062	Heart failure	Mean (SD): 74.5 (9.2)	47.0	United States	Median: 1.8	D	ICD-9
Chuang et al. (2015)	123,300	Hyperlipidemia	Mean: 54.6	49.1	Asia	1–15	D	ICD-9
Hendrie et al. (2015)	974	>70 years	Mean (SD): 76.6 (4.9)	30.3	United States	6.0 ± 2.1	D, AD	DSM-IV, ICD-10
Yang et al. (2015)	3,688	≥65 years with depression	NR	33.9	Asia	Mean: 6.1	D	ICD-9
Liu et al. (2019)	2012	Diabetes mellitus and prostate cancer	Mean: 74.3	100.0	Asia	Mean: 3.5	D	ICD-9
Pan et al. (2018)	9,448	Stroke	Mean (SD): 62.2 (14.7)	55.0	Asia	Median: 7.5	D	ICD-9
Wändell et al. (2018)	12,096	≥45 years with atrial fibrillation	Mean (SD): 72.3 (10.1)	54.4	Europe	5.6 ± 2.5	D	ICD-10
Chang et al. (2019)	100,610	Hyperlipidemia	Mean: 71.7	46.0	Asia	0.5–12	D	ICD-9
Redelmeier et al. (2019)	28,815	≥65 years with cerebral concussion	Mean: 76	38.7	United States	Mean: 3.9	D	ICD-9
Kim et al. (2020)	143,174	≥65 years with ischemic heart disease	Mean: 72.2	40.3	Asia	Mean: 5	D	ICD-10
Lee et al. (2020a)	56,018	>50 years with periodontitis	NR	48.4	Asia	Median: 7.5	D	ICD-9
Lee et al. (2020b)	6,128	40–79 years with hypercholesterolemia	Mean: 66.6	38.6	Asia	Median: 11.7	D, AD	ICD-10
Torrandell-Haro et al. (2020)	288,515	≥45 years	Mean (SD): 67.2 (3.8) in statin users; 66.0 (3.2) in nonusers	45.1	United States	5.1 ± 2.3	D, AD	ICD-9
Zhou et al. (2021)	18,846	≥65 years	Median (25th-75th): 74 (71.6–77.7)	43.6	United States and Australia	Median 4.7	D, AD	DAM-IV
Pham et al. (2022)	39,066	≥65 years	Mean (SD): 74.1 (6.2)	42.4	Canada	Mean: 9	D	CPCSSN case definition
Yang et al. (2022)	33,190	Stroke	Median (25th-75th): 74 (62–83)	48.1	Europe	Mean: 4.8	D	ICD-10
Ren et al. (2024)	104,295	>18 years with heart failure	Mean (SD): 74.2 (13.6)	50.3	Asia	Median: 9.9	D, AD	ICD-9, ICD-10
Jong et al. (2023)	52,139	≥40 years with chronic kidney disease	Median: 65	70.9	Asia	Median: 5.2	D	ICD-9, ICD-10
Kim et al. (2023)	91,018	Atrial fibrillation	Mean: 69.3 in statin users; 67.9 in nonusers	45.4	Asia	Median: 2.1	D	ICD-10
Zhou et al. (2023)	4,207	>40 years with cerebral vascular diseases	Mean (SD): 68.1 (12.0)	55.8	Asia	5.15 ± 3.79	D	ICD-9
Rajan et al. (2024)	4,807	≥65 years	Median (25th-75th): 71.9 (71.6–72.1)	47.0	United States	Median 9.8	AD	NINCDS-ADRDA
Sun et al. (2024)	823,752	≥60 years with diabetes mellitus	Mean: 67.8 for statin users; 67.6 for nonusers	52.8	Asia	3–13	D	ICD-9

3MSE, Modified Mini-Mental State Examination; AD, Alzheimer’s disease; ADAS-Cog, Alzheimer’s Disease Assessment Scale-Cognitive Subscale; CPCSSN, canadian primary care sentinel surveillance network; D, dementia; DSM, diagnostic and statistical manual of mental disorders; F.U., follow-up; ICD, international classification of diseases; MMSE, Mini-Mental State Examination; NINCDS-ADRDA, National Institute of Neurological and Communicative Disorders and Stroke and the Alzheimer’s Disease and Related Disorders Association; NR, not reported; SD, standard deviation.

**FIGURE 2:**
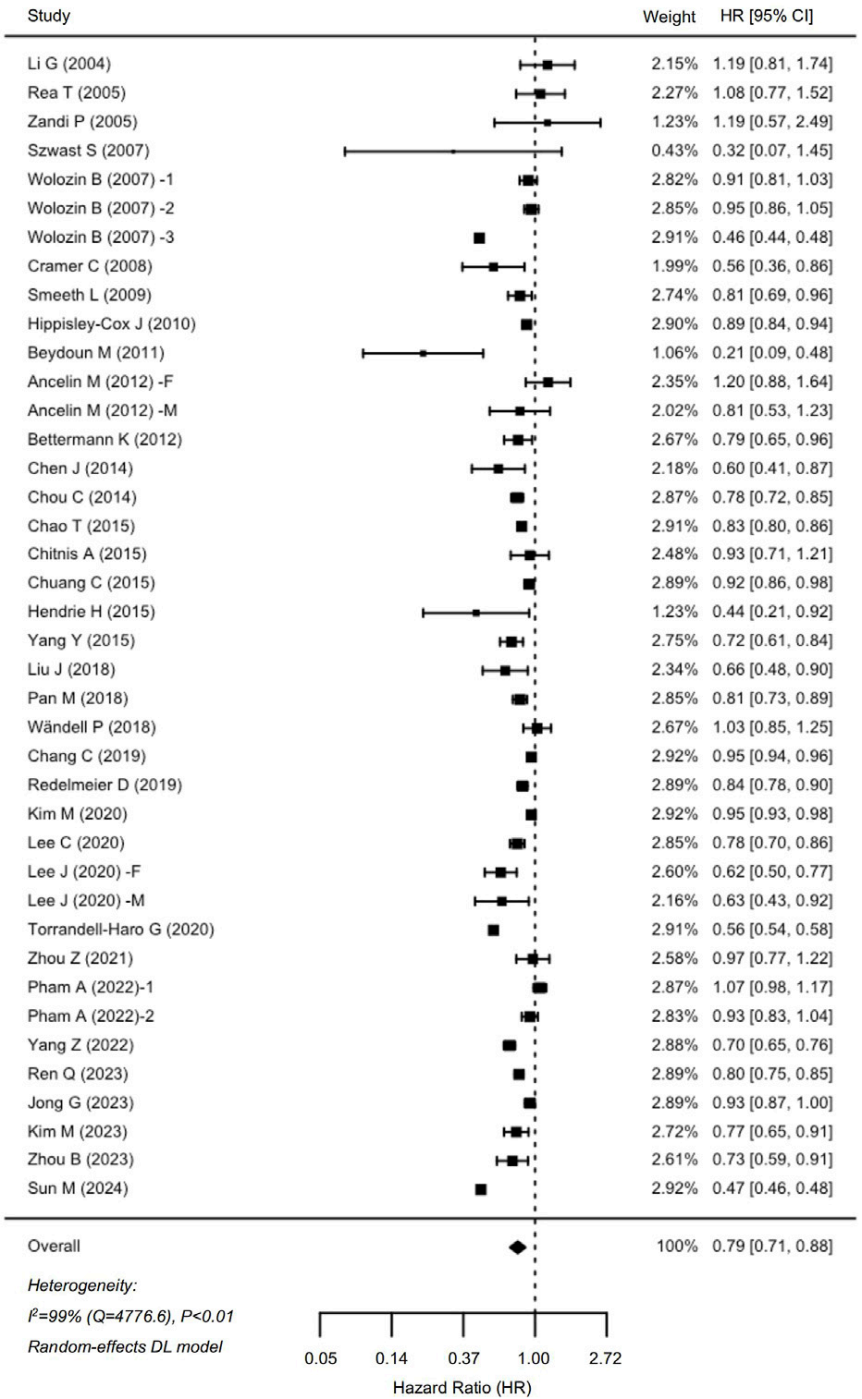
Forrest plot showing effects of statin use on the incidence of dementia. CI, confidence interval.

**FIGURE 3:**
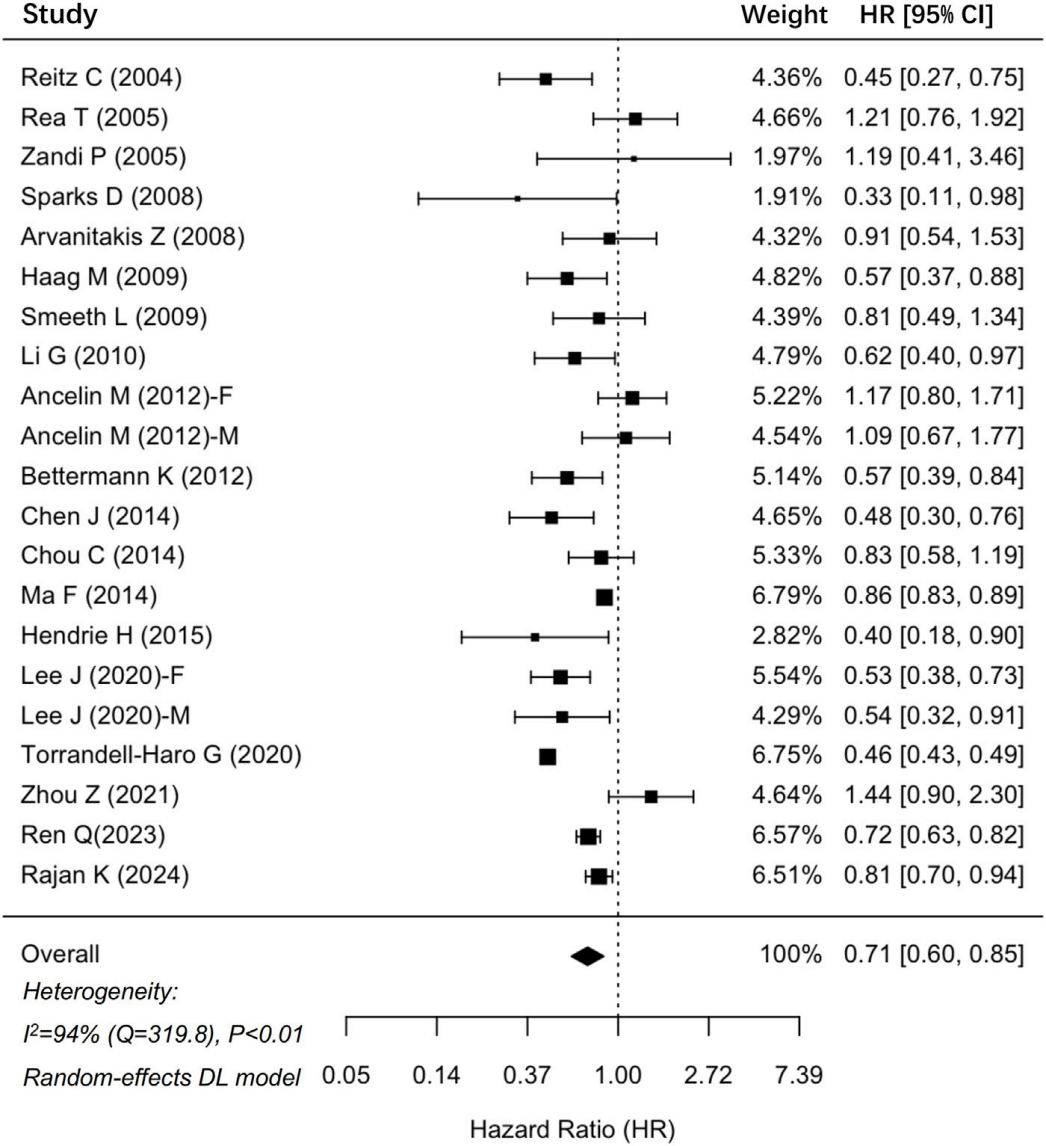
Forrest plot showing effects of statin use on the incidence of Alzheimer’s disease. CI, confidence interval.

In total, 35 cohort studies involving 6,306,043 participants were analyzed for dementia risk (Figure 2). The findings indicated that statin use was associated with a reduced risk of dementia (HR: 0.79, 95% CI: 0.71–0.88). Similarly, an analysis of 19 studies comprising 1,237,341 participants demonstrated a notable 29% decrease in the risk of AD among statin users (HR: 0.71, 95% CI: 0.60–0.85) (Figure 3). The interstudy heterogeneities were substantial for both outcomes, with I^2^ values of 99% for dementia and 94% for AD (Figures 2, 3).

Sensitivity analyses indicated that removing studies with a medium or high risk of bias, conducted before 2010, those with small sample sizes, or those focusing on specific populations did not significantly alter the results (P > 0.05 for all comparisons). However, excluding studies that used non-ICD diagnostic criteria significantly affected the combined risk estimates for dementia (HR: 0.80, 95% CI: 0.80–0.81) and AD (HR: 0.57, 95% CI: 0.45–0.72) (Supplementary Figure S2). Additionally, removal of any single trial did not markedly affect the outcomes. Importantly, interstudy heterogeneities remained significant in our sensitivity analyses (I^2^ > 50% for all sensitivity analyses).

**FIGURE 4:**
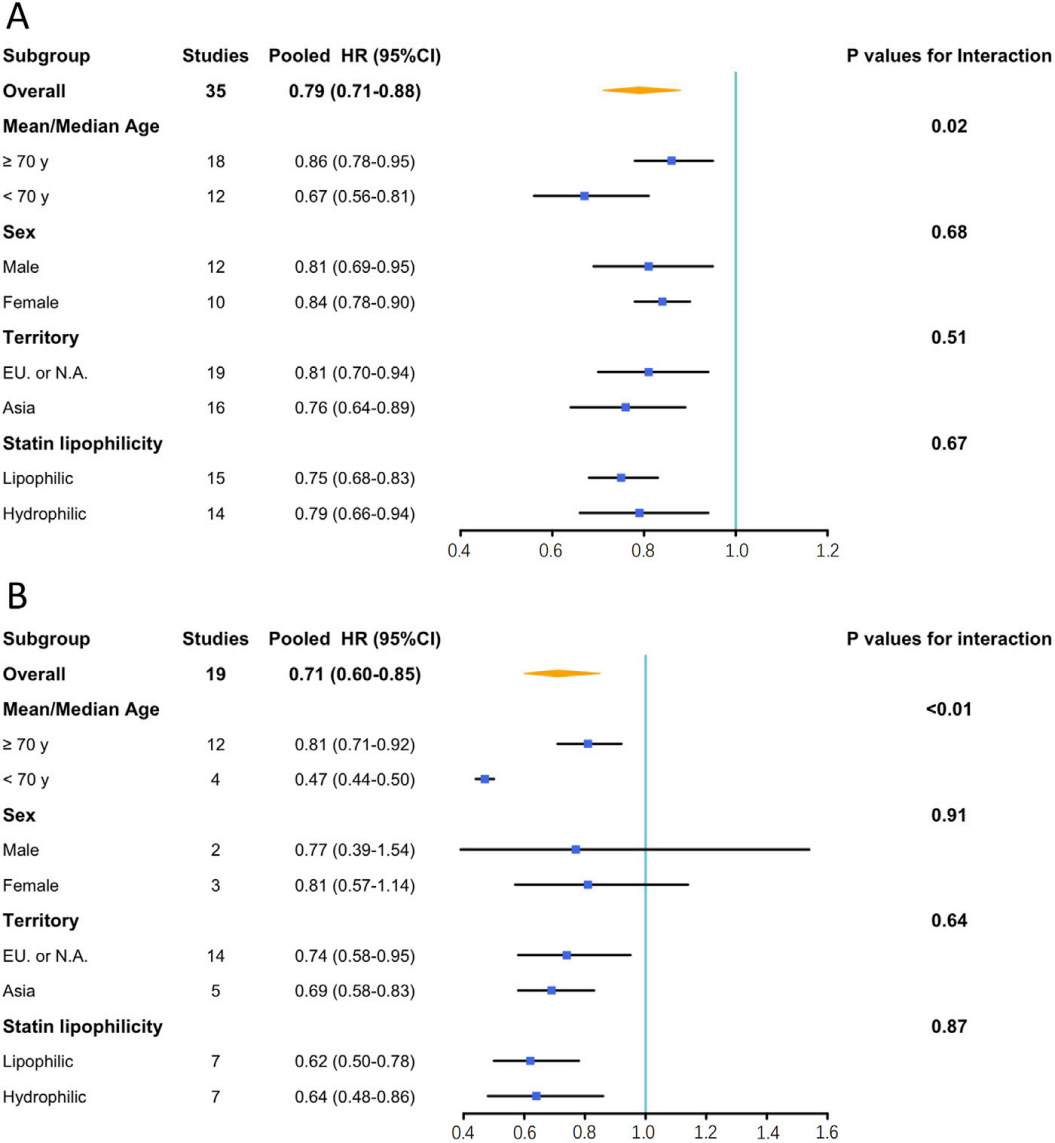
Results of subgroup analysis. **(A)**: Effects of statin on the incidence of dementia; **(B)**: Effects of statin on the incidence of Alzheimer’s disease. CI, confidence interval; EU, Europe; HR, hazard ratio; N.A., North America.

Subgroup analyses demonstrated that compared to studies enrolling participants with a mean/median age over 70 years, those enrolling participants with mean/median age younger than 70 years exhibited greater efficacy of statins in preventing dementia (HR: 0.67, 95% CI: 0.56–0.81 vs. HR: 0.86, 95% CI: 0.78–0.95; P = 0.02) and AD (HR: 0.47, 95% CI: 0.44–0.50 vs. HR: 0.81, 95% CI: 0.71–0.92; P < 0.01). No significant differences were observed in the stratified analyses based on sex, territory, and statin lipophilicity. Figure 4 summarizes the results of the subgroup analyses, and Supplementary Figures S3–S6 present the details of the analyses.

We examined the effects of statin dosage and exposure duration on the incidence of dementia and AD. Due to significant heterogeneity in the definitions of statin dosage and exposure duration among the included studies, pooling the results was deemed inappropriate. Table 2 summarizes the reported effects of statin dosage and exposure duration on dementia and AD from the included studies. Overall, it appears that higher dosages and longer exposure durations of statins further reduce the risk of dementia and AD.

**TABLE 2:** The impact of Intensity and duration of statin on dementia and AD incidence.

Study	Intensity and duration of statin	HR for dementia	HR for AD
Li et al. (2004)	Average daily dose <4 equivalents^a^	1.18 (0.76–1.84)	1.00 (0.54–1.88)
	Average daily dose ≥4 equivalents^a^	1.18 (0.85–3.87)	0.63 (0.21–1.91)
	<4 equivalents/d * 1year	0.93 (0.53–1.65)	0.95 (0.46–1.97)
	≥4 equivalents/d * 1year	1.56 (0.79–3.10)	0.73 (0.26–2.05)
Rea et al. (2005)	<1year	0.98 (0.55–1.74)	1.52 (0.78–2.98)
	1–3 years	1.41 (0.89–2.25)	1.05 (0.49–2.24)
	>3 years	0.74 (0.35–1.57)	1.04 (0.42–2.56)
Zandi et al. (2005)	≤3 years	1.40 (0.49–3.21)	1.19 (0.35–2.96)
	>3 years	0.71 (0.12–2.32)	0.62 (0.03–2.92)
Haag et al. (2009)	≤0.89 DDD	—	0.57 (0.32–1.00)
	>0.89 DDD	—	0.58 (0.32–1.04)
	≤2.9 years	—	0.44 (0.25–0.80)
	>2.9 years	—	0.78 (0.44–1.32)
Chen et al. (2014)	Mean daily dose^b^: <10 mg/d	0.53 (0.34–0.83)	0.38 (0.22–0.67)
	Mean daily dose^b^: 10–20 mg/d	0.68 (0.39–1.17)	0.57 (0.31–1.08)
	Mean daily dose^b^: >20 mg/d	0.80 (0.39–1.64)	0.74 (0.34–1.61)
Chou et al. (2014)	≤1 year	1.07 (0.97–1.19)	—
	1–3 years	0.76 (0.66–0.87)	—
	>3 years	0.35 (0.28–0.43)	—
Chao et al. (2015)	Exposure duration: First quartile	Reference	—
	Exposure duration: Second quartile	0.84 (0.77–0.92)	—
	Exposure duration: Third quartile	0.53 (0.48–0.59)	—
	Exposure duration: Fourth quartile	0.27 (0.25–0.30)	—
Chuang et al. (2015)	Cumulative exposure (per month)-Simvastatin	0.97 (0.96–0.99)	—
	Cumulative exposure (per month)-Fluvastatin	0.97 (0.94–1.00)	—
	Cumulative exposure (per month)-Lovastatin	1.02 (1.00–1.03)	—
	Cumulative exposure (per month)-Atorvastatin	0.98 (0.96–0.99)	—
	Cumulative exposure (per month)-Pravastatin	1.02 (0.99–1.04)	—
	Cumulative exposure (per month)-Rosuvastatin	0.95 (0.92–0.98)	—
Pan et al. (2018)	<1 year	1.25 (1.12–1.39)	—
	1–3 years	0.78 (0.67–0.91)	—
	>3 years	0.28 (0.23–0.35)	—
Chang et al. (2019)	28–365 cDDD	1.90 (1.79–2.01)	—
	366–730 cDDD	1.13 (1.02–1.24)	—
	>730 cDDD	0.44 (0.40–0.49)	—
Redelmeier et al. (2019)	Maximum accepted dosage	0.84 (0.72–0.99)	—
	Lower dosage	0.87 (0.81–0.94)	—
Kim et al. (2020)	Atorvastatin: <1 year	1.13 (1.05–1.22)	—
	Atorvastatin: ≥1 year	0.95 (0.92–0.98)	—
	Simvastatin: <1 year	1.16 (1.05–1.27)	—
	Simvastatin: ≥1 year	0.99 (0.95–1.05)	—
	Rosuvastatin: <1 year	0.95 (0.82–1.11)	—
	Rosuvastatin: ≥1 year	0.81 (0.76–0.86)	—
	Pitavastatin: <1 year	1.05 (0.86–1.28)	—
	Pitavastatin: ≥1 year	0.87 (0.76–0.86)	—
	Pravastatin: <1 year	0.89 (0.72–1.11)	—
	Pravastatin: ≥1 year	0.86 (0.78–0.95)	—
	Fluvastatin: <1 year	0.89 (0.66–1.22)	—
	Fluvastatin: ≥1 year	0.83 (0.69–0.99)	—
	Lovastatin: <1 year	1.09 (0.77–1.55)	—
	Lovastatin: ≥1 year	0.79 (0.62–1.00)	—
Kim et al. (2023)	Low intensity^c^	0.81 (0.69–0.94)	—
	Moderate intensity^c^	0.74 (0.61–0.88)	—
	High intensity^c^	0.67 (0.55–0.80)	—
Sun et al. (2024)	cDDD: First quartile	0.72 (0.69–0.74)	—
	cDDD: Second quartile	0.54 (0.52–0.56)	—
	cDDD: Third quartile	0.38 (0.36–0.40)	—
	cDDD: Fourth quartile	0.20 (0.19–0.21)	—

^a^ 10 mg simvastatin; 20 mg lovastatin; 5 mg atorvastatin.

^b^ Simvastatin or atorvastatin.

^c^ Defined based on 2013 ACC/AHA, guideline on the treatment of blood cholesterol to reduce atherosclerotic cardiovascular risk in adults: a report of the American College of Cardiology/American Heart Association Task Force on Practice Guidelines.

cDDD, cumulative defined daily dose; DDD, defined daily dose.

Visual assessment of the funnel plots (Supplementary Figure S6) and Egger’s tests (P = 0.49 for dementia; P = 0.56 for AD) did not indicate significant publication bias.

## 4 Discussion

Our updated meta-analysis of 42 cohort studies examined the relationship between statin therapy and the incidence of all-cause dementia and AD. Statin use was found to be associated with a significant reduction in the risk of dementia (21%) and AD (29%). These effects varied notably based on the diagnostic criteria used for dementia and AD, with studies using ICD diagnostic criteria showing more pronounced benefits for preventing AD. Subgroup analyses revealed statins provided more protective benefits against both dementia and AD in younger patients. Additionally, although the results could not be pooled owing to significant heterogeneity, most studies indicated that higher statin dosages and longer durations of use were linked to further reductions in the risk of dementia and AD.

Statins are widely recommended globally for both primary and secondary prevention of cardiovascular disease. In 2020, the utilization of statins was estimated at 68.3 defined daily doses (DDDs) per 1,000 individuals aged 40 and above per day (TPD). In high-income regions, this usage has risen to nearly 200 DDDs/TPD (Guadamuz et al., 2022). While statins significantly reduce the incidence and mortality of cardiovascular disease, their potential impact on cognitive function is a pertinent concern. The mechanisms through which statins affect cognitive function are complex (Jamshidnejad-Tosaramandani et al., 2022). The brain is the body’s most cholesterol-rich organ, raising questions about whether lipid metabolism modified by statins could induce structural and functional changes, particularly for lipophilic statins, which can cross the blood-brain barrier and affect brain function independently of circulating cholesterol levels (Goldstein et al., 2023). Some evidence suggests that statins may have a detrimental effect on cognitive function (Rosoff et al., 2022; Strom et al., 2015). Conversely, statins may also improve cognitive function. Vascular dementia, a common type of dementia, is associated with atherosclerosis and may benefit from statin therapy. Additionally, statins have been shown to reduce oxidative stress and inflammation, and improve endothelial function and blood flow (Olmastroni et al., 2022).

To explore the role of statin therapy in the incidence of dementia and AD, several meta-analyses have reviewed and combined results from both case-control and cohort studies, supporting the benefits of statin use in preventing dementia and AD (Olmastroni et al., 2022; Chu et al., 2018). Following a recent meta-analysis, nine new cohort studies involving 1,171,320 patients were published and included in our study (Ren et al., 2024; Kim et al., 2023; Rajan et al., 2024; Zhou et al., 2021; Pham et al., 2022; Yang et al., 2022; Jong et al., 2023; Zhou et al., 2023; Sun et al., 2024). After including these studies, our results align with the recent meta-analysis, which reported a significant risk reduction of 20% for dementia and 32% for AD associated with statin use (Olmastroni et al., 2022). Importantly, considerable heterogeneity was detected among the studies, likely due to differences in study design, diagnostic criteria for dementia and AD, and patient characteristics. To address this, we performed sensitivity and subgroup analyses. For the sensitivity analyses, studies that had a medium or high risk of bias, small sample sizes, were conducted before 2010, involved specific patient populations, or used non-ICD diagnostic criteria were excluded. Interestingly, the diagnostic criteria for dementia and AD significantly influenced the effectiveness of statins. Studies applying the ICD diagnostic criteria suggested that statin therapy offered greater benefits in preventing dementia and AD. These results underscore the importance of unified diagnostic criteria in studies on dementia and AD. A recent study reported that diagnostic criteria significantly influence dementia prevalence, and the agreement between diagnostic criteria was unsatisfied (Wetterberg et al., 2024).

In the subgroup analyses, we investigated whether our results were modified by factors such as patients’ age, sex, region, and statin type. Younger patients (mean/median age ≤70 years) were found to benefit more from statin therapy in preventing future dementia and AD compared with older patients. This finding has important clinical implications and emphasizes the significance of early statin use. Despite of less efficacy, statins still reduce the incidence of dementia and AD in the older adults. Additionally, our analyses showed that the efficacy of statins was consistent across different regions, indicating that ethnicity and socioeconomic factors did not impact the results. Sex and statin lipophilicity did not appear to significantly affect outcomes, which is consistent with previous meta-analyses (Olmastroni et al., 2022). However, it is important to note that these evaluations were constrained by the limited number of studies reporting data stratified by sex and statin lipophilicity.

To explore the effect of intensity and exposure duration of statins on the incidence of dementia and AD. We reviewed included studies that reported relevant findings and found that pooling the results was inappropriate due to variations in the definitions of statin intensity and exposure duration. However, most reported results indicated that longer exposure, higher intensity and cumulative dosage are associated with a lower incidence of dementia and AD (Table 2). These results reinforced the finding of our subgroup analyses that statin therapy achieved a better preventive effect in younger patients, who may receive higher intensity and longer exposure to statins. These results are important, emphasizing the need for adherence to high intensity statin therapy, which is also recommended for preventing cardiovascular disease.

Moreover, the heterogeneity in these studies was not entirely explained by probable study biases. Thus, the heterogeneity is likely attributable to patient-level factors and the heterogeneous nature of dementia and AD. Many unknown factors may modify the effects of statins on cognitive function. Recently, a potential association of statins with a lower risk of incident AD was reported among individuals with the APOE e4 allele, but not in those without APOE e4 allele (Rajan et al., 2024). The results indicate that further investigation of the factors modifying the statin effects on dementia and AD are warranted (Jamshidnejad-Tosaramandani et al., 2022).

### 4.1 Limitation

This study had some limitations. First, there was great heterogeneity among the studies, which might have reduced the reliability of our results. Additionally, this study is a synthesis of observational cohort studies. These studies are prone to bias and confounding factors, both known and unknown, that may not be fully controlled for. Therefore, the results should be interpreted with caution. For example, the observed protective effect might partly stem from the “healthy user bias”, in which individuals receiving preventive treatments, such as statins, are also more likely to engage in other preventive health measures or adopt healthier lifestyles. Despite these limitations, observational cohort studies can offer valuable data on long-term effects and rare adverse outcomes. Future high-quality randomized controlled trials are required to provide more definitive evidence. However, conducting such studies is challenging owing to the slow progression and low incidence of clinical dementia and AD. Recently, the STAREE-Mind Imaging Study (ClinicalTrials.gov Identifier: NCT05586750), a randomized placebo-controlled trial, was initiated to examine the impact of statin use on preventing cerebrovascular decline and neurodegeneration, as detected by magnetic resonance imaging (Harding et al., 2023). Lastly, except for age, the present study did not identify any factors that significantly modified the effects of statins on cognitive function. Given the high heterogeneity in the etiologies of dementia and AD, the effects of statins may vary across different populations. Identifying the populations that benefit most from statin therapy is crucial, and factors such as demographic characteristics, comorbidities, socioeconomic environments, and genetic backgrounds may play a role in this process. Therefore, future studies aimed at identifying these factors are warranted.

## 5 Conclusion

Statin use is associated with a reduced incidence of dementia and AD. Younger patients appear to gain more protective benefits from statins against both dementia and AD. Current evidence suggests that higher intensity and longer exposure to statins are linked to a lower risk of developing dementia and AD, in contrary to concerns about an increased risk.

## Data Availability

The original contributions presented in the study are included in the article/Supplementary Material, further inquiries can be directed to the corresponding author.
