# Supplementary material for: The role of statins in dementia or Alzheimer’s disease incidence: a systematic review and meta-analysis of cohort studies
Source: Front Pharmacol. 2025 Feb 3;16:1473796. doi: 10.3389/fphar.2025.1473796 (PMC11830700; doi:10.3389/fphar.2025.1473796)

**The Role of Statins in Dementia or Alzheimer’s Disease Incidence: A Systematic Review and Meta-analysis of Cohort Studies**

**Supplemental Appendix**

**Table S1.** PRISMA reporting checklist.

**Table S2.** Search Strategy

**Figure S1.** Quality evaluation through the New castle-Ottawa scale.

**Figure S2.** Results of sensitivity analysis.

**Figure S3.** Subgroup meta-analysis of statin use by mean/median age of participants.

**Figure S4.** Subgroup meta-analysis of statin use by sex.

**Figure S5.** Subgroup meta-analysis of statin use by territory.

**Figure S6.** Subgroup meta-analysis of statin use for dementia risk by statin lipophilicity.

**Figure S7.** Funnel plot of study size against logit-transformed proportion for all included studies.

**Table S1.** PRISMA reporting checklist*.

| **Section/topic** | **#** | **Checklist item** | **Reported on section** |
| --- | --- | --- | --- |
| **TITLE** | | | |
| Title | 1 | Identify the report as a systematic review, meta-analysis, or both. | Title |
| **ABSTRACT** | | | |
| Structured summary | 2 | Provide a structured summary including, as applicable: background; objectives; data sources; study eligibility criteria, participants, and interventions; study appraisal and synthesis methods; results; limitations; conclusions and implications of key findings; systematic review registration number. | Abstract |
| **INTRODUCTION** | | | |
| Rationale | 3 | Describe the rationale for the review in the context of what is already known. | Introduction |
| Objectives | 4 | Provide an explicit statement of questions being addressed with reference to participants, interventions, comparisons, outcomes, and study design (PICOS). | Introduction |
| **METHODS** | | | |
| Protocol and registration | 5 | Indicate if a review protocol exists, if and where it can be accessed (e.g., Web address), and, if available, provide registration information including registration number. | NA |
| Eligibility criteria | 6 | Specify study characteristics (e.g., PICOS, length of follow-up) and report characteristics (e.g., years considered, language, publication status) used as criteria for eligibility, giving rationale. | Methods |
| Information sources | 7 | Describe all information sources (e.g., databases with dates of coverage, contact with study authors to identify additional studies) in the search and date last searched. | Methods |
| Search | 8 | Present full electronic search strategy for at least one database, including any limits used, such that it could be repeated. | S.A. |
| Study selection | 9 | State the process for selecting studies (i.e., screening, eligibility, included in systematic review, and, if applicable, included in the meta-analysis). | Methods |
| Data collection process | 10 | Describe method of data extraction from reports (e.g., piloted forms, independently, in duplicate) and any processes for obtaining and confirming data from investigators. | Methods |
| Data items | 11 | List and define all variables for which data were sought (e.g., PICOS, funding sources) and any assumptions and simplifications made. | Methods |
| Risk of bias in individual studies | 12 | Describe methods used for assessing risk of bias of individual studies (including specification of whether this was done at the study or outcome level), and how this information is to be used in any data synthesis. | Methods |
| Summary measures | 13 | State the principal summary measures (e.g., risk ratio, difference in means). | Methods |
| Synthesis of results | 14 | Describe the methods of handling data and combining results of studies, if done, including measures of consistency (e.g., I^2^) for each meta-analysis. | Methods |
| Risk of bias across studies | 15 | Specify any assessment of risk of bias that may affect the cumulative evidence (e.g., publication bias, selective reporting within studies). | Methods |
| Additional analyses | 16 | Describe methods of additional analyses (e.g., sensitivity or subgroup analyses, meta-regression), if done, indicating which were pre-specified. | Methods |
| **RESULTS** | | | |
| Study selection | 17 | Give numbers of studies screened, assessed for eligibility, and included in the review, with reasons for exclusions at each stage, ideally with a flow diagram. | Results, Figure 1 |
| Study characteristics | 18 | For each study, present characteristics for which data were extracted (e.g., study size, PICOS, follow-up period) and provide the citations. | Table 1 |
| Risk of bias within studies | 19 | Present data on risk of bias of each study and, if available, any outcome level assessment (see item 12). | Results, S.A. |
| Results of individual studies | 20 | For all outcomes considered (benefit or harms), present, for each study: (a) simple summary data for each intervention group (b) effect estimates and confidence intervals, ideally with a forest plot. | Results, Figure 2-3 |
| Synthesis of results | 21 | Present results of each meta-analysis done, including confidence intervals and measures of consistency. | Results, Figures 2-3 |
| Risk of bias across studies | 22 | Present results of any assessment of risk of bias across studies (see Item 15). | Results, S.A. |
| Additional analysis | 23 | Give results of additional analyses, if done (e.g., sensitivity or subgroup analyses, meta-regression [see Item 16]). | Results, Figures 4 – 5 |
| **DISCUSSION** | | | |
| Summary of evidence | 24 | Summarize the main findings including the strength of evidence for each main outcome; consider their relevance to key groups (e.g., healthcare providers, users, and policy makers). | Discussion |
| Limitations | 25 | Discuss limitations at study and outcome level (e.g., risk of bias), and at review-level (e.g., incomplete retrieval of identified research, reporting bias). | Limitations |
| Conclusions | 26 | Provide a general interpretation of the results in the context of other evidence, and implications for future research. | Conclusions |
| **FUNDING** | | | |
| Funding | 27 | Describe sources of funding for the systematic review and other support (e.g., supply of data); role of funders for the systematic review. | Acknowledgments |

* From: Moher D, Liberati A, Tetzlaff J, Altman DG, for the PRISMA Group. Preferred reporting items for systematic reviews and meta-analyses: the PRISMA statement. BMJ 2009;339:b2535–b2535.

N.R.: not reported, PRISMA: preferred reporting items for systematic reviews and meta-analyses, S.A.: supplemental appendix.

| **Table S2.** Search Strategy | |
| --- | --- |
| Embase | N |
| ‘dementia’exp or Lewy body or cognitive defect or dement*.mp. or alzheimer*.mp. or (Lewy* adj2 body*).mp. or (chronic adj2 cerebrovascular).mp. or ("organic brain disease" or "organic brain syndrome").mp. or (cerebr* adj2 deteriorat*).mp. or (cerebral* adj2 insufficient*).mp. | 522,478 |
| statin:ab,ti OR statins:ab,ti OR atorvastatin:ab,ti OR cerivastatin:ab,ti OR fluvastatin:ab,ti OR lovastatin:ab,ti OR pravastatin:ab,ti OR simvastatin:ab,ti OR lipitor:ab,ti OR baycol:ab,ti OR lescol:ab,ti OR mevacor:ab,ti OR altocor:ab,ti OR pravachol:ab,ti OR lipostat:ab,ti OR zocor:ab,ti OR mevinolin:ab,ti OR compactin:ab,ti OR fluindostatin:ab,ti OR rosuvastatin:ab,ti | 107518 |
| #3 #1 and #2 | 1855 |
| Web of Science |  |
| [(dement* OR alzheimer* OR AD OR VCI OR VaD OR "vascular cognitive impairment" OR "lew* bod*" OR CADASIL OR cognit*) (Abstract) and TOPIC: (statin* OR atorvastatin OR cerivastatin OR fluvastatin OR lovastatin OR pravastatin OR simvastatin OR lipitor OR barcol OR leocol OR megacor OR autocor OR pravach OR lipostar OR zokor OR mevinolin OR compactin OR fluidostatin OR rosuvastatin)](http://webofscience-clarivate-cn-s.webvpn.zju.edu.cn:8001/wos/alldb/summary/6896539b-a687-48cd-a7da-55311b0d9b4f-f4fdbca2/relevance/1) | 316 |
| Pubmed |  |
| #1 (("hydroxymethylglutaryl coa reductase inhibitors"[Pharmacological Action] OR "hydroxymethylglutaryl coa reductase inhibitors"[MeSH Terms] OR ("hydroxymethylglutaryl coa"[Title/Abstract] AND "reductase"[Title/Abstract] AND "inhibitors"[Title/Abstract]) OR "hydroxymethylglutaryl coa reductase inhibitors"[Title/Abstract] OR "statin"[Title/Abstract] OR "statins"[Title/Abstract] OR "statin s"[Title/Abstract] OR "statine"[Supplementary Concept] OR "statine"[Title/Abstract] OR "statines"[Title/Abstract]) OR ("simvastatin"[MeSH Terms] OR "simvastatin"[Title/Abstract] OR "zocor"[Title/Abstract] OR "simvastatin s"[Title/Abstract] OR "simvastatins"[Title/Abstract]) OR ("fluvastatin"[MeSH Terms] OR "fluvastatin"[Title/Abstract] OR "lescol"[Title/Abstract]) OR ("lovastatin"[MeSH Terms] OR "lovastatin"[Title/Abstract] OR "lovastatine"[Title/Abstract] OR "mevacor"[Title/Abstract] OR "lovastatin s"[Title/Abstract]) OR ("atorvastatin"[MeSH Terms] OR "atorvastatin"[Title/Abstract] OR "atorvastatine"[Title/Abstract] OR "lipitor"[Title/Abstract] OR "atorvastatin s"[Title/Abstract]) OR ("rosuvastatin calcium"[MeSH Terms] OR ("rosuvastatin"[Title/Abstract] AND "calcium"[Title/Abstract]) OR "rosuvastatin calcium"[Title/Abstract] OR "crestor"[Title/Abstract] OR "rosuvastatin"[Title/Abstract])) | 74217 |
| #2 ("cognitive dysfunction"[MeSH Terms] OR "cognitive dysfunction"[Titile/Abstract] OR "cognitive decline"[ Titile/Abstract] OR "cognitive impairment"[ Titile/Abstract] OR "dementia"[MeSH Terms] OR "dementia" Titile/Abstract] OR "dementias"[ Titile/Abstract] OR "alzheimes"[ Titile/Abstract] OR "alzheimer disease"[MeSH Terms] OR "alzheimer disease"[ Titile/Abstract] OR "alzheimer"[ Titile/Abstract] OR "alzheimers"[Titile/Abstract] OR "Alzheimer’s"[ Titile/Abstract]) | 234220 |
| #4 #1 and #2 | 1058 |
| CENTRAL |  |
| #1 atorvastatin or cerivastatin or fluvastatin or lovastatin or pravastatin or simvastatin or lipitor or baycol or lescol or mevacor or altocor or pravachol or lipostat or zocor or mevinolin or compactin or fluindostatin or rosuvastatin | 14036 |
| #2 statin or statins | 11461 |
| #3 [Hydroxymethylglutaryl‐CoA Reductase Inhibitors] explode all trees | 5040 |
| #4 #1 or #2 or #3 | 19936 |
| #5 MeSH descriptor: [Dementia] explode all trees | 9444 |
| # 6 (dement*):ti,ab,kw or (alzheimer*):ti,ab,kw or ("lewy* bod*") or ("cerebr* deteriorat*") or ("cerebr* deteriorat*") or | 26181 |
| #7 #5 or #6 | 26543 |
| #8 #4 and #7 | 243 |
| Clinicaltrials.gov ([www.clinicaltrials.gov](http://www.clinicaltrials.gov/)) |  |
| (statins OR statin OR simvastatin OR lovastatin OR pravastatin OR fluvastatin OR atorvastatin OR rosuvastatin) AND (elderly OR cognition OR cognitive OR dementia OR alzheimer OR alzheimers) | 76 |

**Figure S1.** Quality evaluation through the New castle-Ottawa scale


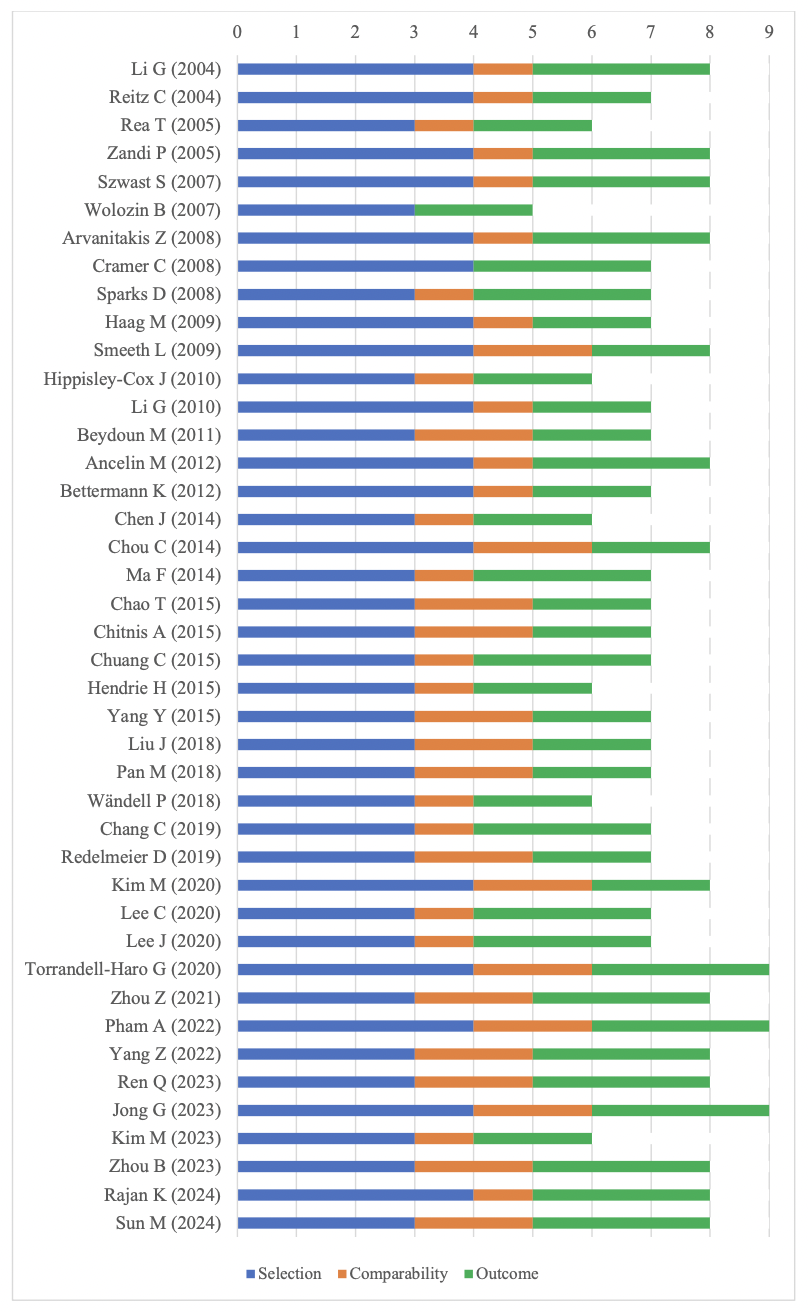


**Figure S2.** Results of sensitivity analysis. CI: confidence interval; HR: hazard ratio; ICD: international classification of diseases. * :P value for testing the null-hypothesis of homogeneity between the included and excluded studies.

**
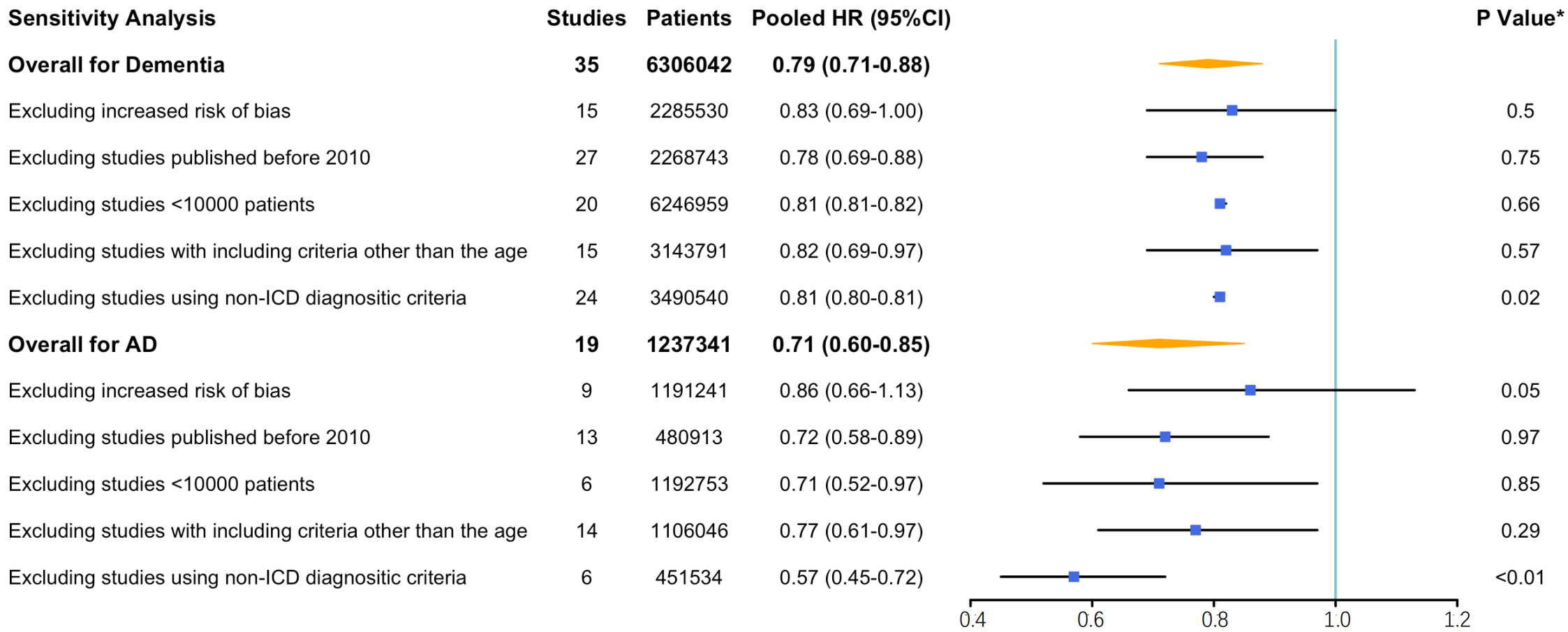
**

**Figure S3.** Subgroup meta-analysis of statin use for dementia risk (A) and for Alzheimer’s disease (B) by mean/median age of participants.

**(A)** **
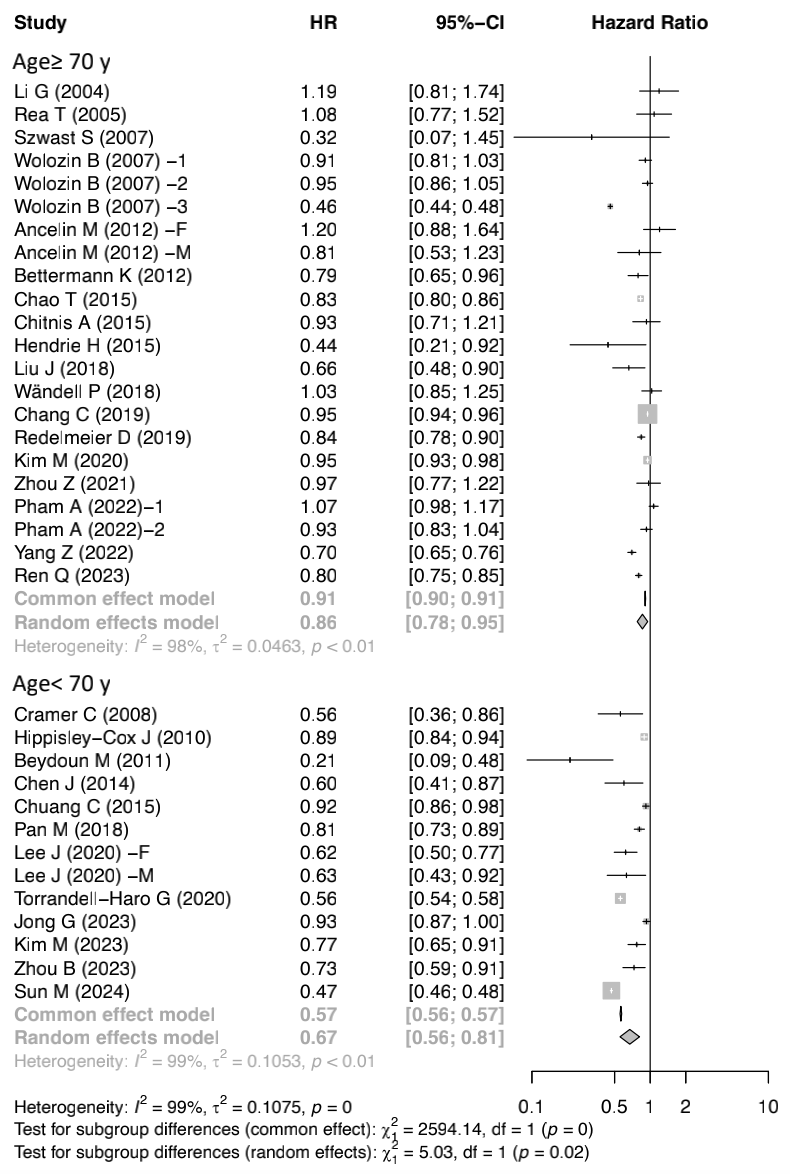
**

**(B)
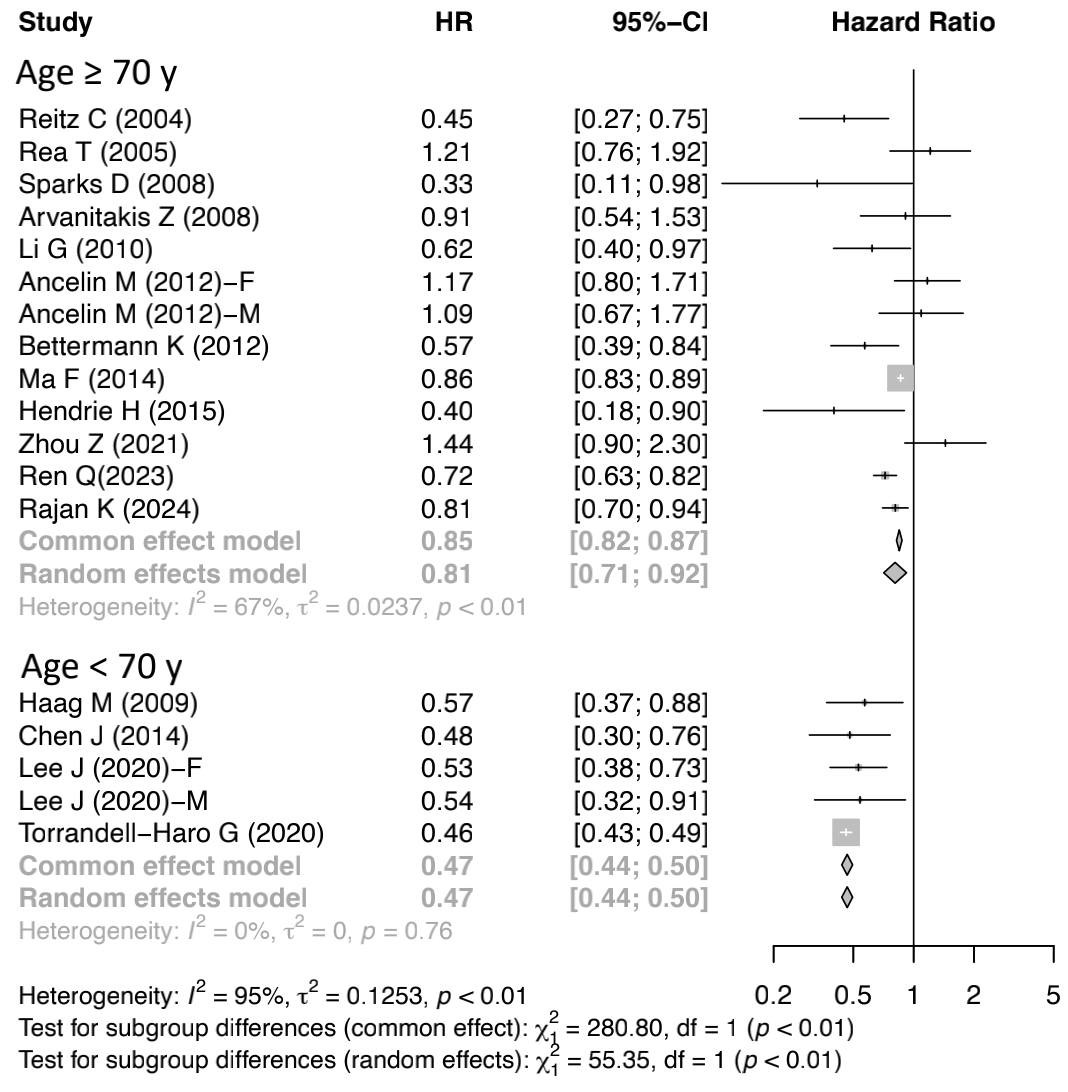
**

**Figure S4.** Subgroup meta-analysis of statin use for dementia risk (A) and for Alzheimer’s disease (B) by sex.

**(A)**
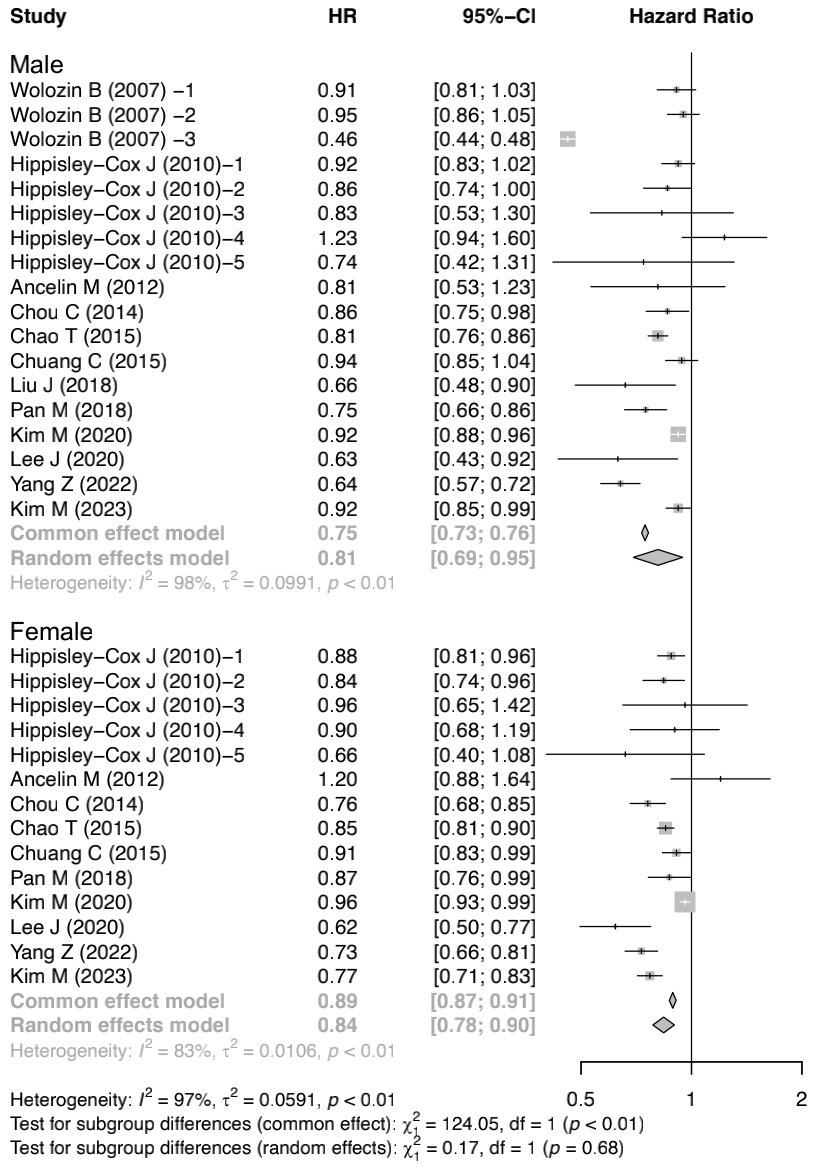


**(B)
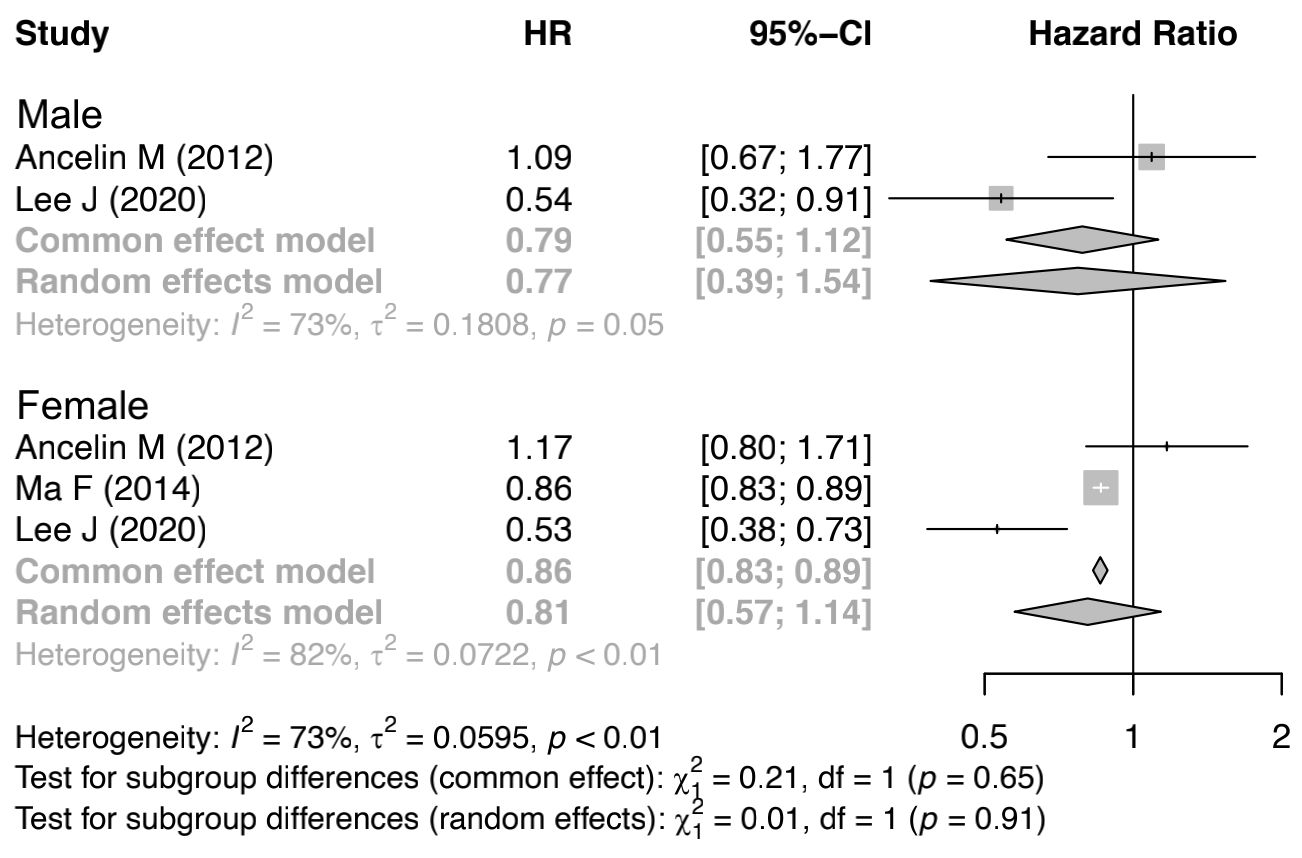
**

**Figure S5.** Subgroup meta-analysis of statin use for dementia risk (A) and for Alzheimer’s disease (B) by territory.

**(A)**

**
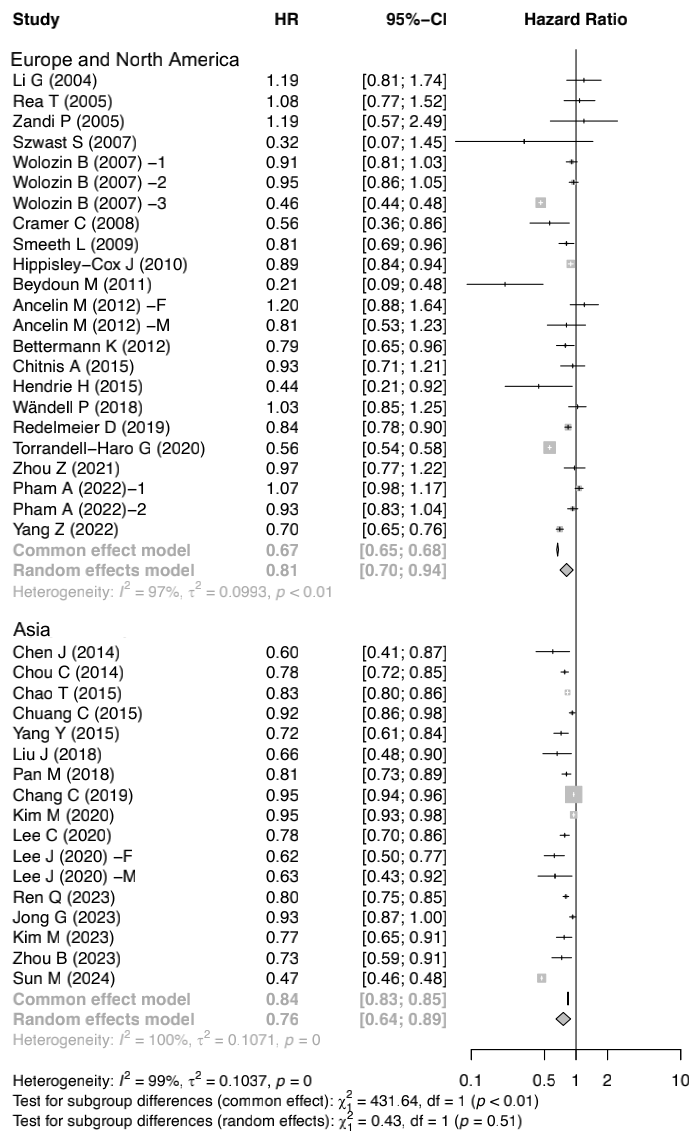
**

**(B)**

**
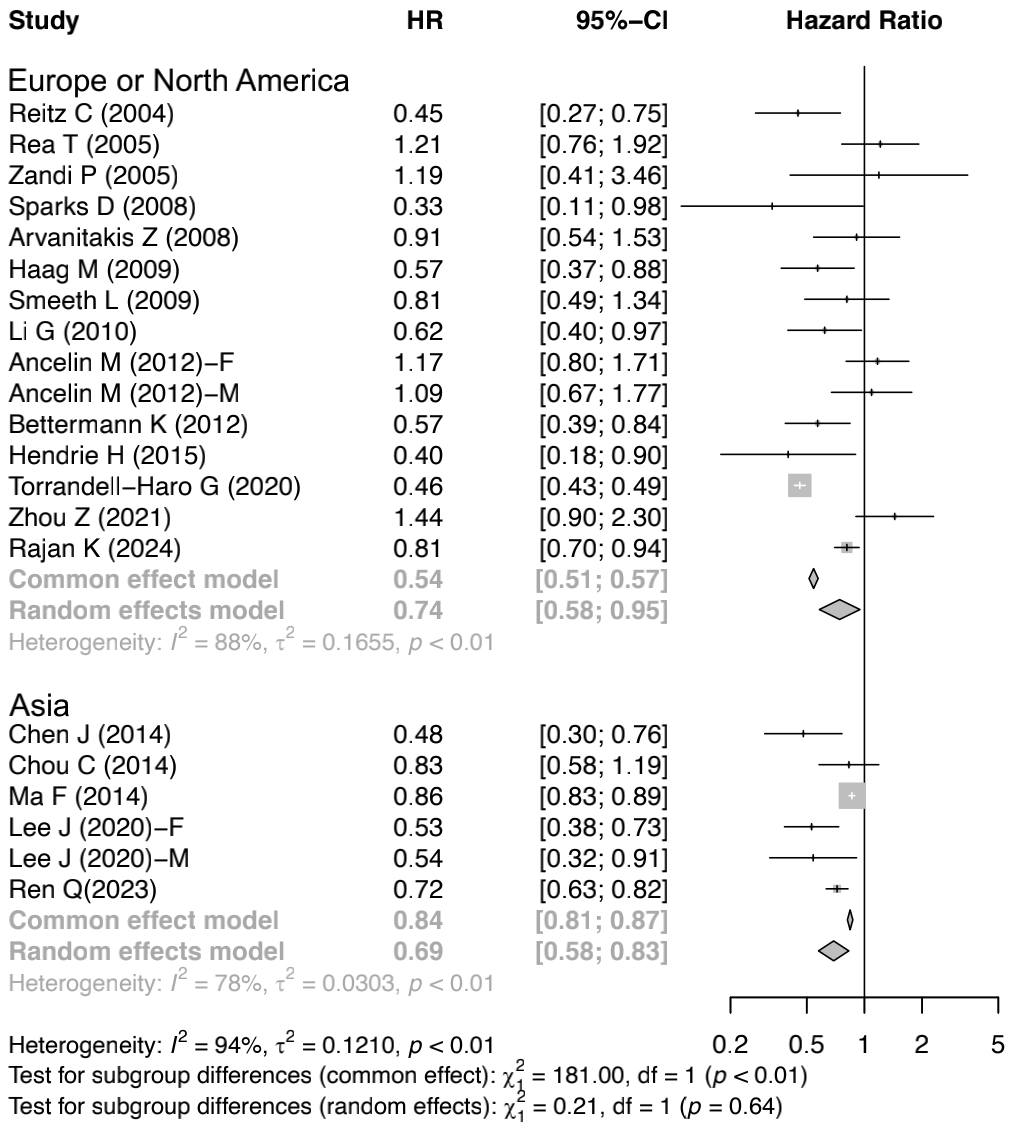
**

**Figure S6.** Subgroup meta-analysis of statin use for dementia risk (A) and for Alzheimer’s disease (B) by statin lipophilicity.

**(A)**


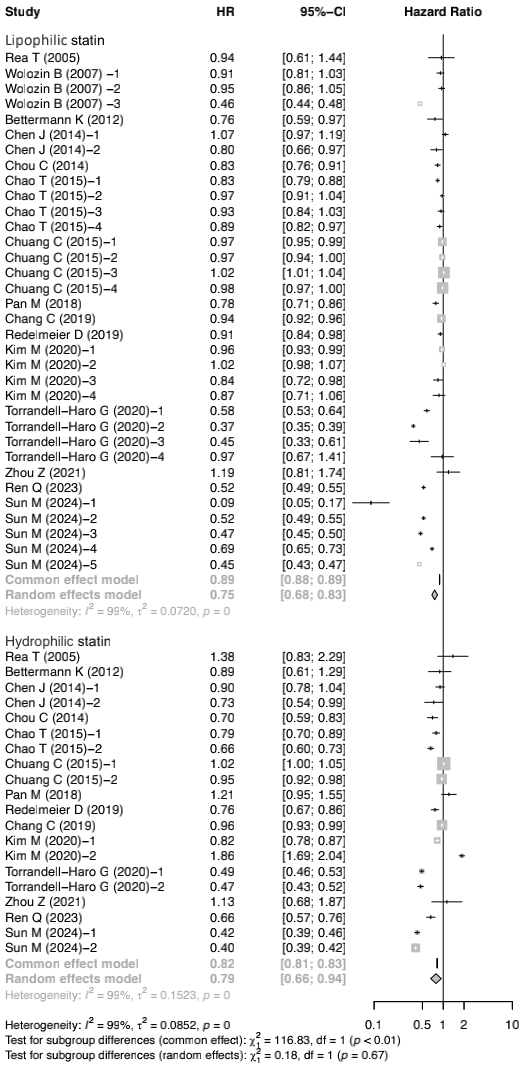


**(B)**


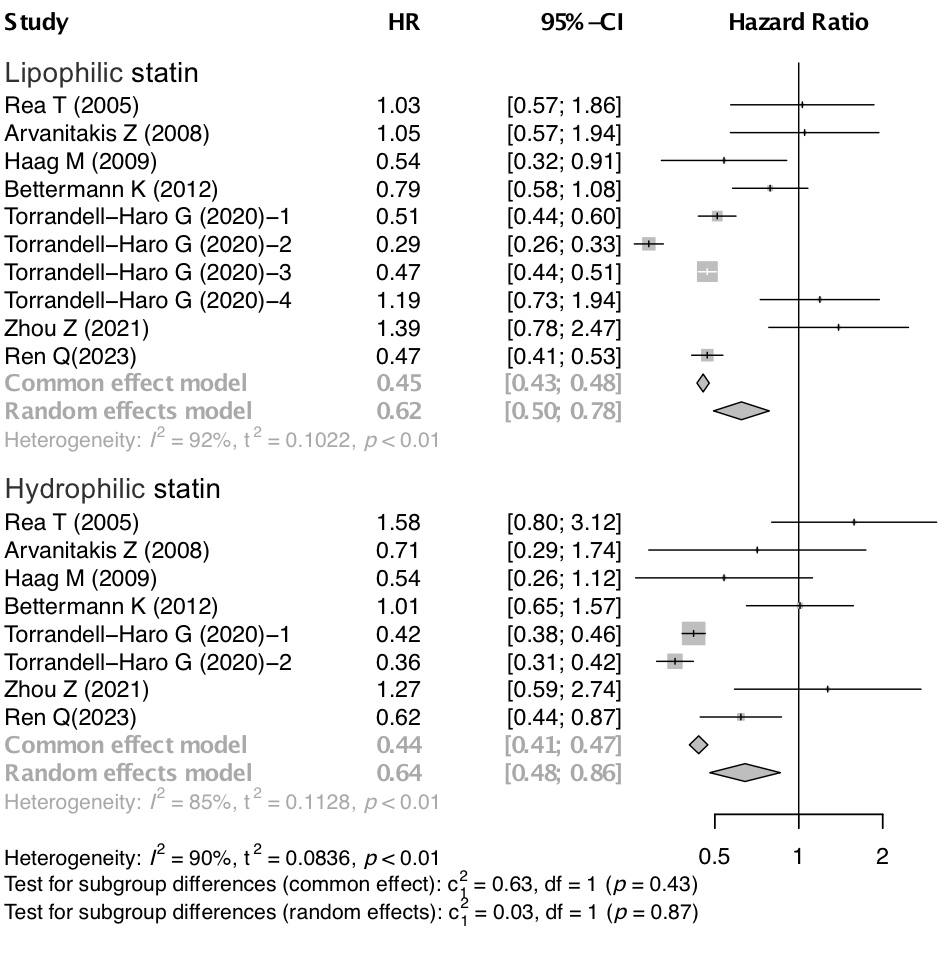


**Figure S7.** Funnel plot of study size against logit-transformed proportion for all included studies. (A) dementia; (B) Alzheimer's disease.


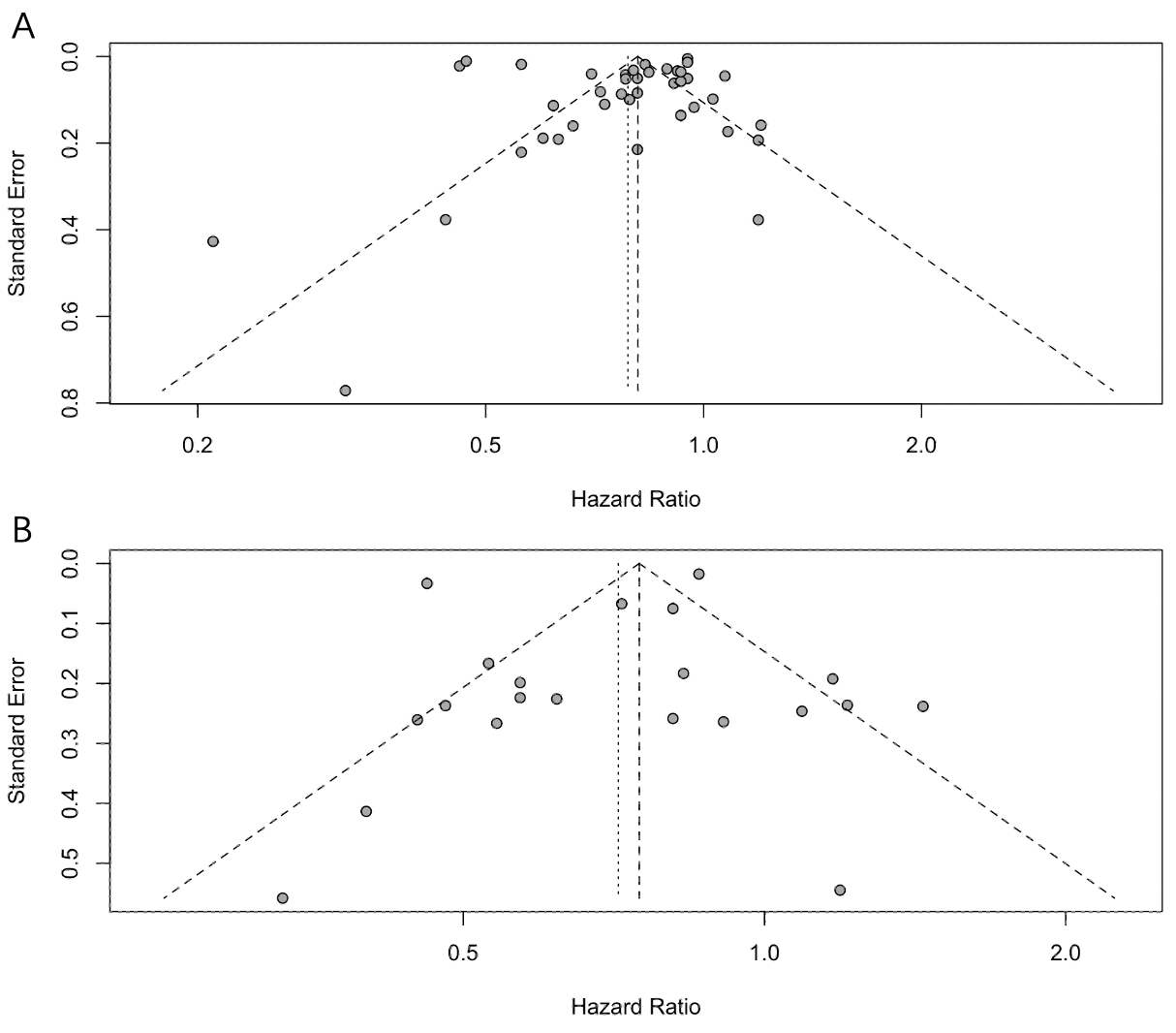

Supplement: Supplementary file 1 [file DataSheet1.docx]
